# One-Pot Reactive Melt Recycling of PLA Post-Consumer Waste for the Production of Block Copolymer Nanocomposites of High Strength and Ductility

**DOI:** 10.3390/polym14173642

**Published:** 2022-09-02

**Authors:** Kalyanee Sirisinha, Supa Wirasate, Chakrit Sirisinha, Noppasorn Wattanakrai

**Affiliations:** 1 Department of Chemistry, Faculty of Science, Mahidol University, Bangkok 10400, Thailand; 2 Rubber Technology Research Centre (RTEC), Faculty of Science, Mahidol University, Nakhon Prathom 73170, Thailand

**Keywords:** poly(lactic acid), block copolymer, nanocomposites, tensile properties, recycling, transesterification

## Abstract

Post-consumer waste recycling is a crucial issue for building a sustainable society. However, mechanical recycling of poly(lactic acid) (PLA) often reduces the performance of the recycled material because PLA has a strong tendency to degrade during reprocessing. Therefore, it is of great interest to develop an effective recycling method to improve the mechanical performance of this material. This paper presents a one-pot melt process for turning PLA waste into a biodegradable block copolymer and its high strength and ductility composite. The process was conducted in a melt-mixer through a transesterification of PLA with poly(ethylene glycol) (PEG) or poly(propylene glycol) (PPG) as a soft component and clay as reinforcement. Effects of soft component content and sequence of clay addition on the mechanical performance of the prepared materials were focused. The results showed the successful preparation of PLA-based multiblock copolymers of high molecular weights (~100 kDa). Both virgin PLA and recycled source could serve as the starting material. PEG was more efficient than PPG in providing an intense improvement of PLA ductility. The nanocomposite of intercalated structure yielded nearly 100 times higher elongation at break (E_b_ = 506%) than the starting PLA (E_b_ = 5.6%) with high strength of 39.5 MPa and modulus of 1.4 GPa, considering the advantages of clay addition. Furthermore, the products with a broadened range of properties can be designed based on the ratio of PLA and soft component, as well as the organization and spatial distribution of clay in the copolymer matrices.

## 1. Introduction

Poly(lactic acid) (PLA) has become an economically viable material due to its high potential for applicability in various areas, such as in biomedical, agricultural, or packaging fields. The global production capacity of PLA was around 400,000 tons in 2021 [1]. The demand for PLA in different industrial areas has increased as a consequence of the increasing environmental concerns and the depletion of crude oil for the production of traditional oil-based plastics. With its rising usage, more PLA will end in conventional post-consumer plastic waste streams. Although PLA is a biodegradable plastic, the degradation of PLA is slow in the environment. In soil or domestic composters, degradation can take up to a year with temperatures around 20 °C [2,3]. PLA may ultimately break down into its constituent parts (carbon dioxide and water) in a controlled industrial composting environment, requiring temperatures around its glass transition temperature (~60 °C) and high relative humidity [4,5]. In short, the main differences between domestic and industrial composters are temperature and relative humidity. However, degradation is not the only or the most effective treatment method for biodegradable PLA waste. The recycling of post-consumer waste as a secondary raw material, therefore, plays a major role in making the use of natural resources more efficient and reducing the negative impact of plastics on the environment.

Several methods for managing the PLA wastes include composting and depolymerization. According to life cycle analysis, mechanical recycling has more environmental benefits because of its relatively simple process and low pollution risk [6]. For most conventional petroleum-based plastics, such as polypropylene (PP) and polyethylene (PE), mechanical recycling has been studied quite in-depth [7]. With growing market volumes of PLA, there have been ongoing efforts to study the effect of reprocessing and recycling on the performance of PLA. Beltran and co-authors [8] found that mechanical recycling has a limited impact on the structure, thermal stability, and mechanical properties of PLA and suggested that the recycled PLA could be reused for applications such as packaging. However, adverse effects of mechanical recycling on the performance of PLA were reported in the literature [9]. The studies of Zenkiewicz et al. on the effect of repeated extrusion of neat PLA showed that the melt flow rate (MFR) values of the reprocessed samples steadily increased with increased extrusion cycles. The reductions of tensile and impact strength of the reprocessed materials were then resulted [9]. Pillin et al. [10] observed a substantial reduction of the tensile strength and elongation at break of PLA after repeated injection molding whereas the modulus of PLA was hardly changed. Similarly, the studies of Zhao et al. on the recyclability of 3D-printing PLA showed that the polymer could not be reprocessed beyond two cycles without severe loss of properties and viscosity [11].

Several strategies are proposed for increasing the properties of recycled PLA. For example, a specific multifunctional chain extender was added to the PLA in order to limit or inhibit the degradation of the PLA during processing, with the consequent improvement of the thermo-mechanical and rheological properties of the polymer [12,13]. Another approach concerned the possible use of additives that act by restoring the molecular weight of the PLA as affected by processability loss due to thermo-degradation phenomena [14,15]. Several authors achieved improvements in the mechanical and thermal properties of PLA by adding small amounts of nanofillers [16,17,18,19]. Using nanographene in combination with PLA has shown a reduction in the degradation rate of the material [16]. Clay nanoparticles have also been widely investigated as reinforcements for PLA. The addition of organically modified montmorillonite led to the improvement of the mechanical, thermal, and gas barrier properties of a PLA matrix [17,18,19].

Copolymerization of PLA with soft segment is among the effective ways for producing higher toughness PLA, apart from plasticization by small molecules or polymeric plasticizers, and melt-blending with flexible polymers or elastomers. PLA copolymers with a wide range of properties can be designed based on the innate properties of each constituent, the ratio of PLA and soft phase, and the organization of the repeating units [20,21,22,23,24,25]. A traditional method for producing PLA copolymer is ring-opening polymerization (ROP) of lactide monomer or direct condensation of lactic acid, with soft components such as polycaprolactone (PCL), and poly(ethylene glycol) (PEG), etc. The reaction is traditionally performed under reduced pressure for many hours, and a large amount of solvent is required in the process [21,22,23]. Our previous study demonstrated a solventless method for preparing PLA copolymers using a high molecular weight PLA as a feed material [24,25]. The transesterification of PLA and polyol was undertaken in a melt state in the presence of a catalyst. A chain extender was then introduced for the purpose of linking the short-chain copolymers to gain a high molecular weight PLA copolymer.

The present work expands the melt-transesterification approach for the production of copolymer nanocomposites based on PLA, intending to achieve a biodegradable material of balanced strength, stiffness, and ductility in a one-pot process. In this investigation, the starting PLA materials were those of commercially available grade PLA in pellet form and PLA regrind from post-consumer PLA disposable dome lid for cold drinking cups, which is the most common application of PLA in the household area. First, the PLA block copolymers and their nanocomposites containing clay filler were prepared in a melt-mixer. Next, nuclear magnetic resonance spectroscopy (NMR) and gel permeation chromatography (GPC) were employed to analyze the structure and molecular weight of the prepared block copolymers. X-ray diffraction (XRD) and transmission electron microscope (TEM) were used to investigate the morphology, especially the dispersion and spatial distribution of clay in the composite samples. Finally, tensile test was conducted to evaluate the effects of copolymer types and contents, and the sequence of clay filler addition on the mechanical performance of the products. Furthermore, the degradation behaviors of the PLA products were analyzed.

## 2. Materials and Methods

### 2.1. Materials

Commercial grade PLA, Ingeo™ 2003D, was from Natureworks^®^ LLC, Minnetonka, MN, USA. The post-consumer PLA samples used in the study were disposable clear dome lids for cold drinking cups. The post-consumer wastes were ground and subjected to a washing process. Prior to processing, the washed materials were dried at 60 °C overnight in a vacuum oven. Poly(ethylene glycol) (PEG) with a molecular weight (M_w_) of 4000 g mol^−1^ was purchased from Ajax Finechem Pty Ltd., and poly(propylene glycol) (PPG) of similar molecular weight was from Acros Organics. Tetrabutyl titanate (TBT) and hexamethylene diisocyanate (HDI) were purchased from Sigma-Aldrich Co., LLC, Missouri, USA. Phenolic antioxidant (Irganox 1010) was from BASF, Ludwigshafen, Germany. The clay used was a commercial organoclay montmorillonite (MMT) Cloisite 30B that was modified with an alkyl quaternary ammonium salt, from Southern Clay Products Inc, Gonzales, TX, USA.

### 2.2. Preparation of PLA Block Copolymers and Copolymer/Clay Composites

In contrast to the known procedure for the preparation of PLA-based block copolymers combining ROP of lactide monomer and controllable radical polymerization, PLA block copolymers of this study were synthesized via a transesterification reaction between a high molecular weight PLA (either virgin PLA pellets or PLA regrinds) and a polyol (PEG or PPG) in a melt state. The synthesis strategy started with a melt-transesterification of PLA with a polyol in the presence of a catalyst to form short-chain block copolymers, followed by chain extension to obtain long-chain multiblock copolymer products.

The melt reaction process was performed in a HAAKE PolyLab internal mixer equipped with cam-blade rotors. The PLA samples were melt-blending with PEG (or PPG) at 170 °C for 10 min using a rotor speed of 50 rpm. The 0.74 wt% TBT as a catalyst was then added, and the reaction was continued for 5 min. After this, the HDI chain extender was introduced. After 3 min of reaction, the PLA-based multiblock copolymers were obtained. The simple blends of PLA and polyol (plasticized PLA) were prepared, and the neat PLA was also thermally treated under the same conditions as above for comparison.

In the case of copolymer nanocomposites, 3 wt% of clay was introduced to the system as a reinforcing filler. The effect of processing sequences of clay addition was investigated. In Method A, PLA block copolymer was first prepared, and clay was then compounded with the copolymer in an internal mixer at 170 °C for 5 min. The composite was named “copolymer composite”. In Method B, PLA, PEG, and clay were thoroughly mixed in an internal mixer at 170 °C for 5 min, and then a transesterification reaction was performed by adding catalyst and chain extender to the formulation. The resulting material, in this case, was denoted as “reactive copolymer composite”. The obtained copolymers and copolymer composites were then compression-molded at 170 °C into specimens with dimensions specific to each test. The specimens were stored in a desiccator at 23 °C prior to testing.

### 2.3. Characterizations and Testing

Nuclear magnetic resonance spectroscopy (NMR) was used to analyze the structure of the PLA block copolymers. ^1^H NMR spectra of the purified samples were recorded on an NMR spectrometer (Bruker 500 MHz, Madison, WI, USA) operated at 25 °C with 25,000 scans to obtain high-resolution spectra. Deuterated chloroform (CDCl_3_) was used as a solvent. Gel permeation chromatography (GPC) instrument (Waters 1515, Milford, MA, USA) equipped with RI detector was used to determine the average molecular weights and polydispersity (PDI) of the block copolymers using tetrahydrofuran (THF) as an eluent (at 40 °C) and polystyrene (PS) as a standard.

XRD analysis was performed on a Malvern Panalytical Empyrean X-ray diffractometer with CuK_α_ radiation (*λ* of 0.1540 nm) at a generator voltage of 45 kV and a generator current of 40 mA. The interlayer spacing (*d*_001_) of clay was calculated using Bragg’s law (*λ = 2d sin θ*). TEM was carried out on a Phillips TECNAI 20 transmission electron microscope using an acceleration voltage of 100 kV. Ultrathin samples for TEM were prepared using a Leica Ultracut R ultramicrotome.

Tensile testing was performed using an Instron Model 5566 Universal Tensile tester (Canton, MA, USA). The test was carried out at 23 °C, 50% RH according to ASTM D882 (gauge length of 25.4 mm) with a crosshead speed of 50 mm.min^−1^. At least five specimens were used for averaging. The correlation between stress and strain was studied. Young’s modulus, tensile strength, and elongation at break were determined.

Hydrolytic degradation test was conducted on square specimens of 10.0 × 10.0 mm^2^ with a thickness of 1.0 mm. Each specimen was immersed in 50 mL of 0.1 M pH 7 sodium phosphate buffer containing 2 wt% sodium azide (NaN_3_) antimicrobial substance. The tests were performed at 70 °C for different durations, after which the percentage of weight loss was determined using the following equation.

(1) $$Weight\;loss\;\left(\%\right)=\frac{w_{i}-w_{f}}{w_{i}}\times 100$$

where, *w_i_* is initial weight of sample before subjected to testing and *w_f_* is final weight of sample after testing.

## 3. Results and Discussion

### 3.1. PLA-Based Block Copolymers: Structure and Properties

**Figure 1:**
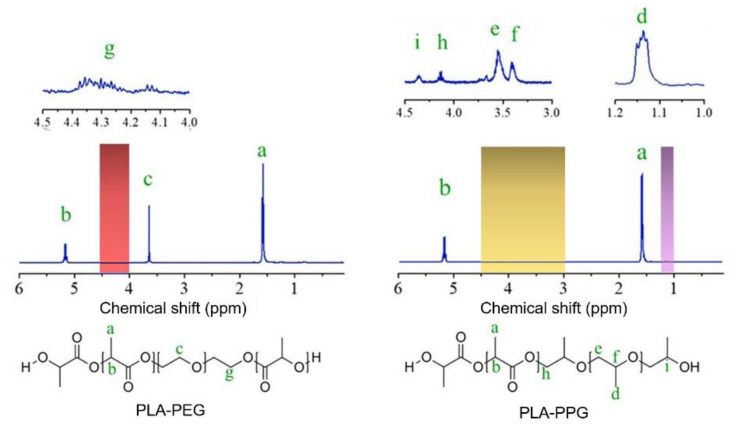
^1^H NMR spectrum of PLA-PEG and PLA-PPG copolymers.

Virgin PLA pellets were used as a starting material for producing PLA-based block copolymers, i.e., PLA-PEG and PLA-PPG, via a melt-transesterification process. The contents of PEG and PPG initially added were 20 wt%. The chemical structures of the two copolymers characterized by ^1^H NMR are given in Figure 1. The signals at 1.60 and 5.20 ppm belong to the methyl protons (a, -CH_3_) and methine protons (b, -CH) of the PLA unit, respectively. The PEG unit exhibits a characteristic signal of methylene protons (c, -CH_2_) at 3.60 ppm [26,27], while the PPG unit shows a signal of methyl protons (d, -CH_3_), methylene protons (e, -CH_2_), and methine protons (f, -CH) at 1.14, 3.55 and 3.40 ppm, respectively. The signals from 4.20 to 4.30 ppm correspond to the methylene protons (g, -CH_2_) of PEG that are attached to the PLA unit, while those belonging to PPG are observed at 4.15 ppm. The ^1^H NMR signal at a chemical shift of 4.35 ppm (-CH, i) of the PLA-PPG sample is assigned to the attached secondary hydroxyl protons of PPG. The molecular weights obtained by GPC are shown in Table 1. The PLA-PEG block copolymer exhibits the M_w_ of approximately 1.3 × 10^5^ g mol^−1^ whereas the M_w_ of PLA-PPG copolymers is around 1.1 × 10^5^ g mol^−1^. Compared to the starting PLA (M_w_~1.7 × 10^5^ g mol^−1^), the PLA-based copolymers yield slightly lower molecular weights and broader molecular weight distributions. NMR and GPC results thus confirm that the PLA-PEG and PLA-PPG multiblock copolymers of high molecular weights were successfully prepared by a melt-transesterification process employing PLA polymer as a starting material.

Table 2 contains the mechanical properties of a range of plasticized PLA and PLA-based block copolymers according to soft segment type and content. Neat PLA exhibits a high tensile strength of 64.73 MPa and modulus of 2.27 GPa with a very low elongation at break of only 6%. This is as expected, as the PLA resembles clear polystyrene in which it is stiff and brittle, and consequently needs modification for most practical applications (i.e., plasticization and/or copolymerization to increase its flexibility). Evidence of the plasticizing effect of PPG and PEG is clearly seen in Table 2, where the addition of either PPG or PEG makes PLA less brittle and enhances its extensibility significantly. PEG displays better plasticizing abilities than PPG. The plasticizing effect is amplified by increasing the amount of plasticizer in the blends. However, there is a limit of the plasticizer content in practice. The addition of plasticizer beyond the limit not only leads to a deterioration in mechanical strength and stiffness, but also facilitates phase separation. The separation of plasticizer causes increased stiffness and decreased drawability of the plasticized PLA. Moreover, the plasticizer may leach out and migrate from the bulk to the product surfaces thereafter. [28,29]. In some particular areas including food contact packaging, plasticizer migration from the packaging materials is not allowed.

An important factor in the commercial development of ductile PLA is the ability to maintain its ductility during functional use. Copolymerization of PLA with soft segment offers plasticizer-free solutions and, thus, is a beneficial approach for modifying its properties. It is clear from Table 2 that both PLA copolymers with a soft component in the structure yield higher elongation than the neat PLA. For example, the copolymer with 10%PEG (PLA-PEG) exhibits more than 100 times higher elongation (E_b_~600%) than the starting PLA (E_b_~6%) together with a strength of 30.25 MPa and modulus of 952.31 MPa. Despite having an equal molecular weight of 4000, PEG is more efficient than PPG in providing an intense improvement of PLA ductility. The primary hydroxyl terminated groups of PEG can react readily with the ester functions of PLA chains, enabling the achievement of a multiblock PLA-PEG copolymer with high ductility.

### 3.2. Nanocomposites of PLA Block Copolymer and Clay

**Figure 2:**
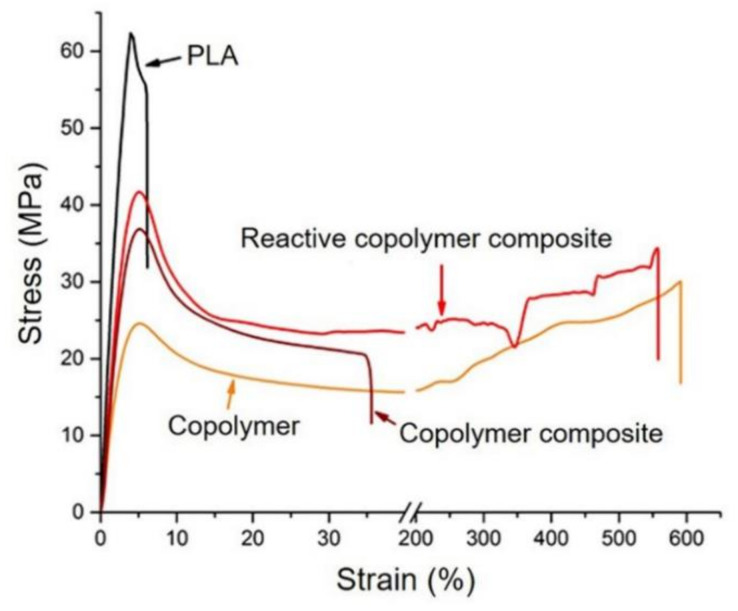
Stress-strain curves of PLA, PLA-PEG (80–20) block copolymer, block copolymer composite (from Method A), and reactive copolymer composite (from Method B).

Based on the results gained from the tensile study, PEG with its relatively high efficiency was selected as a soft block component for enhancing the ductility of PLA. MMT clay was introduced to the formulation to compensate the reduced stiffness and strength of PLA caused by the presence of soft PEG component in the system, aiming at obtaining a nanocomposite with a good balance of strength, stiffness, and ductility. In this part, post-consumer PLA regrind was used as a feed material. PEG contents were varied at 10 and 20 wt%. The effects of sequences of clay addition on the structure and mechanical properties of the composites were focused. Stress-strain dependencies for the materials studied are illustrated in Figure 2, and the average values of tensile strength, Young’s modulus, and elongation at break are summarized in Table 3.

In contrast to the neat PLA showing brittle failure at a low strain, the PLA-PEG copolymer exhibits dramatically increased ductility, and undergoes strain-hardening with the elongation at break of approximately 600%. The presence of strain hardening indicates a very intense effect of PEG on increased segmental mobility, thus facilitating the plastic deformation of the block copolymers upon tensile drawing. Other notable factors influencing the mechanical performance of PLA-based materials are the content of soft segment and the processing sequence of clay addition. Interestingly, the reactive copolymer composite prepared from Method B is capable of undergoing strain hardening, showing an extraordinarily high magnitude of elongation at break of >500% whereas the copolymer composite prepared from Method A shows a much lower elongation at break (80%).

The tensile data summarized in Table 3 reveal a similar trend of mechanical property changes for both composite series prepared from virgin PLA feedstock and post-consumer PLA regrind. The notably high strain at break value of 441.79% along with tensile strength of 35.98 MPa and Young’s modulus of 969.68 MPa are observed in the reactive copolymer composite sample with 20 wt% PEG soft segment using PLA regrind as a feed material. The magnitudes of tensile strength and Young’s modulus show an increasing trend when decreasing the content of PEG soft segment in the formulation. For example, a highly ductile nanocomposite with an elongation of ~500% and high modulus of 1417.70 MPa could be achieved from a one-pot reactive melt process using PLA regrind copolymerized with 10 wt% PEG in the presence of clay. The results also indicate that only a small amount of clay nanofiller (3 wt%) is needed for a great improvement in the stiffness and strength of PLA-based block copolymer. Better mechanical performance of the reactive copolymer nanocomposite deserves further investigation. In polymer/clay nanocomposites, great enhancement of mechanical properties and thermal stability are believed to result from synergistic interactions between the high surface area of clay galleries and the polymer, combined with the specific constitution of the system via intercalation and/or exfoliation phenomena [30,31,32]. In this study, the nanocomposite morphological characteristics were investigated by XRD and TEM.

**Figure 3:**
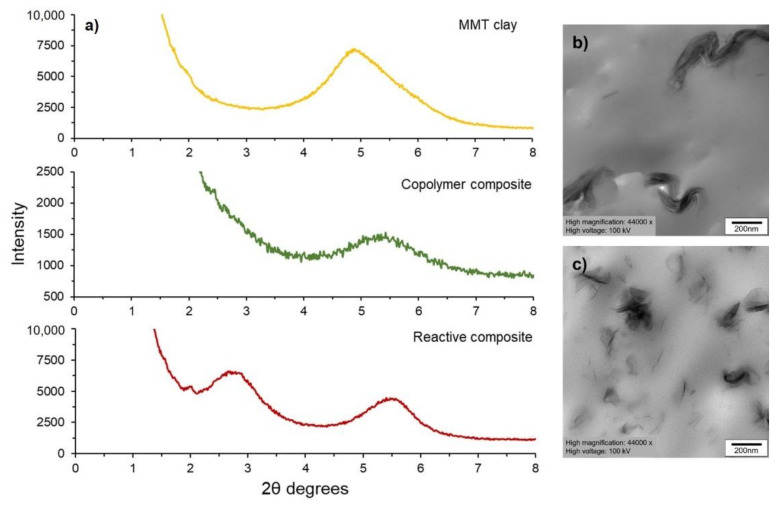
XRD patterns for MMT clay, copolymer composite, and reactive copolymer composite (**a**), and TEM images of copolymer composite (**b**), and reactive copolymer composite (**c**).

The XRD patterns, shown in Figure 3a, reveal the structure of MMT clay (Cloisite 30B) and clay in the nanocomposites. The MMT clay exhibits a 2θ at 4.8 degree characterized by an interlayer distance (*d*_001_) of 1.8 nm, which is in line with the previous work [33,34,35,36]. By monitoring the position, shape, and intensity of the basal reflections from the distributed clay layers, the nanocomposite structure (intercalated or exfoliated) could be identified. The Cloisite 30B peak (2θ = 4.8 degree) could be observed in the XRD patterns of the two nanocomposites prepared by different techniques, suggesting that the MMT layered configuration existed. However, the appearance of a new basal reflection at a lower 2θ angle of 2.7 degree is found in the reactive copolymer composite, reflecting a larger interlayer distance (*d*_001_ = 3.3 nm) of clay in the material and the occurrence of an intercalated structure together with a partial preservation of MMT layered structure. The nanocomposite structure is further confirmed by the TEM micrographs of Figure 3b,c, revealing the presence of MMT layered configuration and more compacted sheets in the composite prepared from the conventional method A (Figure 3b). In contrast, the reactive composite presents more separated clay sheets and a better clay distribution (Figure 3c).

**Figure 4:**
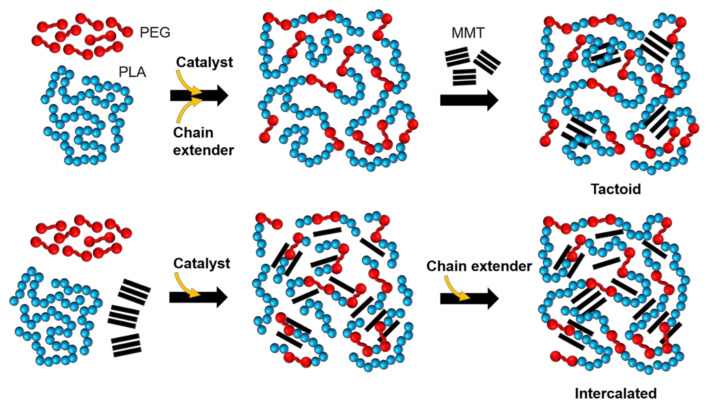
Schematic illustration of melt-copolymerization and nanocomposite formation.

Schematic representation of the melt-copolymerization and nanocomposite formation has been presented in Figure 4. In the case of the nanocomposite prepared from Method A, a long-chain multiblock copolymer was formed in the first stage and then clay was added to a highly viscous melt, resulting in the clay arrangement in the form of tactoids- consisting of several stacked silicate layers. For the reactive nanocomposite, clay was thoroughly mixed with the polymers prior to performing the melt-copolymerization reaction. This allows the movement of short-chain block copolymer into the clay galleries, giving the sliding of the silicate sheets and increasing the separation between the clay layers, thus promoting an intercalation and partially exfoliated structure.

**Figure 5:**
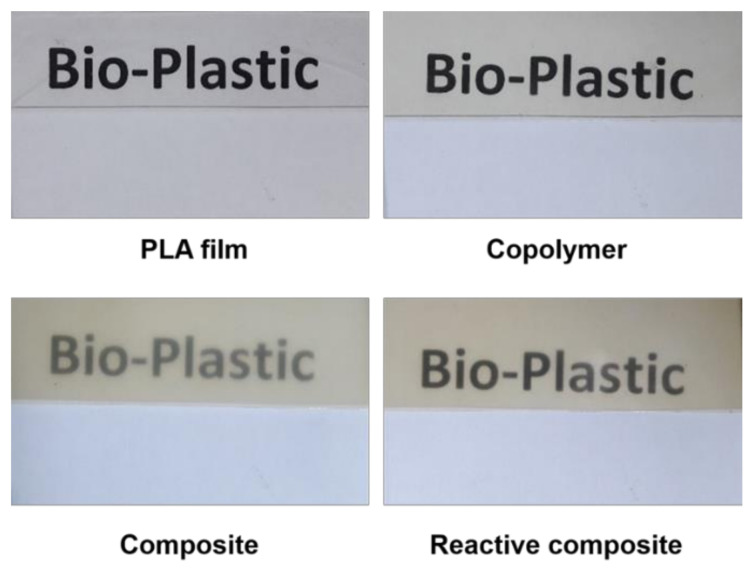
Photographs of 0.4 mm thick films of PLA, PLA-PEG block copolymer, copolymer composite, and reactive copolymer composite.

Visual examination of the 0.4 mm thick molded samples shows some opacity for the composite obtained from Method A, whereas the reactive composite prepared by Method B and the unfilled block copolymer are more transparent (Figure 5). The opacity of the composite prepared from Method A resulted from the light scattering on the filler particles. In turn, the transparency of the reactive nanocomposite confirms good dispersion and spatial distribution of clay within the polymer matrix.

Table 4 provides the tensile data of various bio-based and petroleum-based polymers, together with the similar PLA-based block copolymers prepared by different synthesis routes [37,38,39,40,41,42]. From the analysis of the mechanical data reported in Table 4, it is evident that the tensile properties of PLA-PEG block copolymers reported in the literatures are quite different. This different behavior is mainly related to the content of soft segment in the polymer chains and the molecular weight of the prepared copolymers. The increase of molecular weight seems to offer an enhanced tensile strength of the copolymers. With the high M_w_ of about 100 kDa, the PLA-PEG multiblock copolymers of this study yield high tensile strength (25–28 MPa), modulus (524–740 MPa), and elongation at break (>500%). Comparable values of tensile properties were observed by Mei et al., reporting a tensile strength of 32.6 MPa and elongation of about 500% for the PLA-PEG multiblock copolymer with a M_w_ of 145 kDa. However, this sample exhibited a modulus of only 28.4 MPa [38]. Biodegradable poly(butylene adipate-co-terephthalate) (PBAT, Ecoflex from BASF) is characterized by high elongation at break (>300%), with strength and modulus of 18.8 and 30 MPa, respectively [40]. A relatively higher modulus (450 MPa) is reported for poly(butylene succinate) (BioPBS, FZ91PD from PTT MCC Biochem), with a tensile strength of 33.3 MPa and elongation at break of 330% [41]. Considering the benefits obtained with a nanoscale distribution of clays within the polymer matrix in terms of improved mechanical properties at low filler content, PLA-based block copolymer composites of this study exhibit comparable properties to some petroleum-based polymers grade widely used in packaging applications such as polypropylene [42]. Our research, therefore, provides a facile method for making environmentally sustainable products of good mechanical performance from PLA post-consumer waste that would serve useful industrial markets such as disposable packaging and agricultural films. The process is economically feasible, as it can be carried out in the melt without solvent, thus providing a low environmental impact.

**Figure 6:**
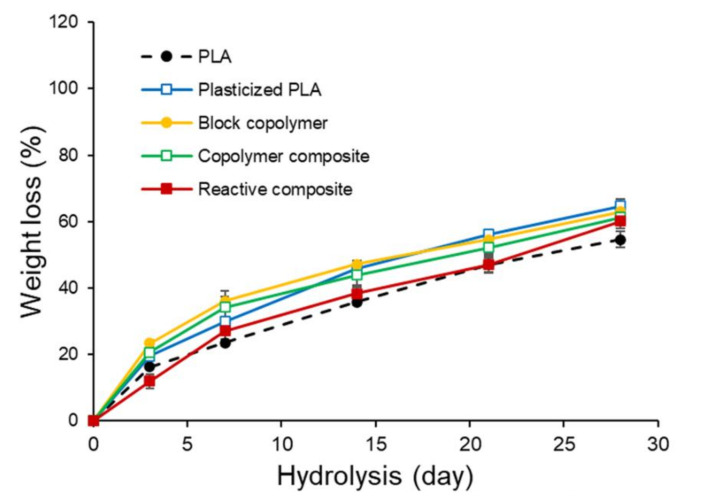
Evolution of weight loss with increasing hydrolysis time for PLA, plasticized PLA, block copolymers and composite.

The primary degradation mechanism of PLA is hydrolysis, catalyzed by temperature, followed by bacterial attack on the fragmented residues. The end result of the process is carbon dioxide and water. In aqueous systems, the hydrolysis of PLA can be achieved by bulk or surface erosion [43]. The rate of degradation is influenced by the rate of water diffusion which in turn depends on the original molecular weight of the sample, crystallinity, sample dimensions, pH, and temperature. In this study, the hydrolysis of PLA, its block copolymers, and copolymer composites was carried out at 70 °C for up to 30 days. Regardless of the sample types, the degradation rate increases with increasing the hydrolysis time (Figure 6). The presence of PEG as either a plasticizer or a soft segment in the polymer chains elevates the rate of PLA hydrolysis traced by weight loss. PEG has a considerable affinity to water so its copolymer might have an increased hydrophilicity [44,45]. In addition, the PLA-PEG block copolymer exhibits a slightly lower molecular weight than the starting PLA. Both factors, therefore, are probably responsible for the higher degradation rate found in the block copolymer products.

The copolymer nanocomposites containing clay are hydrolyzed more readily than the unmodified PLA but still more difficult than the unfilled copolymer. The weight loss percentages after 30-day hydrolysis of PLA, its copolymer, and plasticized sample were 55, 63, and 65, respectively. The incorporation of clay gives somewhat reduction in the weight loss of the copolymers. The composite structure (microcomposite or intercalated nanocomposite) has been reported to play a determining role in the hydrolytic degradation process of PLA [46]. In this study, the reactive nanocomposites with their intercalated structure showed slightly lower weight loss value than those of tactoid structure.

## 4. Conclusions

PLA-based multiblock copolymers and their composites were prepared from post-consumer waste via melt-transesterification using TBT as a catalyst and HDI as a chain extender. It was evident by NMR and GPC analysis that copolymerization of PLA in the melt state did not induce a dramatic drop of PLA molecular weight. The block copolymers with M_w_ of approximately 10^5^ g mol^−1^ were successfully prepared. Despite having an equal molecular weight of 4000, PEG was more efficient than PPG in enhancing the ductility of PLA. However, the presence of soft component reduced both tensile strength and modulus of the samples. The tensile results indicated that only a small amount of clay (3 wt%) was needed to drastically enhance the strength and stiffness of the copolymers. The localization and spatial distribution of clay in the copolymer matrices as well as the overall properties of the composites depended strongly on the PLA/PEG ratio and the sequence of clay addition. For the reactive composites where clay was thoroughly mixed with the polymers before performing the copolymerization reaction, the TEM investigation revealed the separation between the clay layers consistent with *d* spacing obtained from XRD. Due to the nanometer-range dispersion of clay layers, this material showed very high elongation at break of 500% and high modulus of 1.41 GPa with high transparency. In addition, the copolymer composites exhibited a slightly higher rate of hydrolysis than the PLA. Our research, therefore, provided an effective method for turning PLA post-consumer waste into sustainable and value-added products with appropriate properties to be used in industrial markets such as packaging and agricultural films.

## Figures and Tables

**Table 1:** Molecular parameters for the virgin PLA, PLA regrind, and PLA-based copolymers.

Properties	PLA Virgin	PLA Regrind	PLA-PEG Copolymer	PLA-PPG Copolymer
Weight average molecular weight (M_w_)	1.7 × 10^5^	1.4 × 10^5^	1.3 × 10^5^	1.1 × 10^5^
Number average molecular weight (M_n_)	8.1 × 10^4^	7.4 × 10^4^	5.0 × 10^4^	4.8 × 10^4^
Polydispersity index (PDI)	1.96	1.83	2.78	2.29

**Table 2:** Tensile strength, Young’s modulus, and elongation at break of various plasticized PLA and PLA-based block copolymers.

Soft Segment [%]	Plasticized PLA			PLA-Based Block Copolymer		
	Strength [MPa]	Modulus [MPa]	Elongation [%]	Strength [MPa]	Modulus [MPa]	Elongation [%]
PLA	64.73 ± 3.50	2269.03 ± 119.48	5.69 ± 1.12			
PPG						
10	40.74 ± 0.98	1971.66 ± 143.00	25.48 ± 60.40	45.40 ± 1.21	1811.50 ± 71.65	5.60 ± 0.16
15	34.83 ± 1.83	1893.64 ± 123.18	49.90 ± 16.01	33.89 ± 1.16	1613.92 ± 46.55	36.27 ± 3.67
20	32.31 ± 1.09	1804.29 ± 102.49	53.66 ± 10.34	25.23 ± 0.51	1418.90 ± 41.20	14.04 ± 4.24
PEG						
10	52.88 ± 0.57	1657.66 ± 34.34	25.71 ± 0.40	30.25 ± 1.98	952.31 ± 53.59	606.31 ± 27.71
15	41.37 ± 2.05	1095.88 ± 40.30	528.17 ± 17.82	26.90 ± 1.49	546.33 ± 32.25	644.52 ± 31.28
20	35.23 ± 1.61	549.12 ± 10.19	478.51 ± 20.81	24.92 ± 0.71	523.95 ± 19.94	620.14 ± 17.88

**Table 3:** Tensile properties of a range of PLA-PEG block copolymers, copolymer composites, and reactive copolymer composites.

Samples	PEG Content [%]	Strength [MPa]	Modulus [MPa]	Elongation [%]
PLA virgin	-	64.73 ± 3.50	2269.03 ± 119.48	5.69 ± 1.12
Block copolymer	20	24.92 ± 0.71	523.95 ± 19.94	620.14 ± 17.88
Copolymer composite	20	36.60 ± 1.20	1330.02 ± 60.58	87.54 ± 3.28
Reactive copolymer composite	20	42.32 ± 1.13	1410.45 ± 70.58	562.15 ± 42.39
PLA regrind	-	59.30 ± 1.70	2242 ± 201.02	6.15 ± 0.92
Block copolymer	20	27.42 ± 1.16	740.04 ± 31.2	587.20 ± 45.41
Copolymer composite	20	34.02 ± 2.17	954.29 ± 53.59	102.50 ± 17.00
Reactive copolymer composite	20	35.98 ± 1.47	969.68 ± 58.92	441.79 ± 20.87
PLA regrind				
Block copolymer	10	26.25 ± 1.98	952.31 ± 53.59	572.31 ± 29.17
Copolymer composite	10	37.60 ± 0.71	1328.74 ± 64.67	35.24 ± 3.28
Reactive copolymer composite	10	39.49 ± 1.30	1417.70 ± 71.16	506.15 ± 42.39

**Table 4:** Tensile data of various bio-based and petroleum-based polymers, together with the similar PLA-based block copolymers prepared by different synthesis routes.

System	Type of Copolymer	M_w_ [kDa]	PLA/PEG Ratio [wt/wt]	Strength [MPa]	Modulus [MPa]	Elongation [%]	Ref.
PLA-PEG copolymer (M_w_ of PEG = 4000)	multiblock	95–130	80/20	25–28	524–740	580–620	This work
Copolymer composite (with 3 wt% clay)	multiblock	N/A	80/20	34–37	954–1330	85–102	
			90/10	37.6	1328	35	
Reactive copolymer composite (with 3 wt% clay)	multiblock	N/A	80/20	36–42	970–1410	500	
			90/10	39.5	1418	506	
PLA-PEG copolymer synthesized from ring-opening polymerization of lactide in the presence of PEG	diblock	32.9	79/21	7.0	320	49	[37]
	triblock	57.5	78/22	7.0	225	134	
	multiblock	145	48/52	32.6	28.4	546	[38]
	multiblock	61.1	52/48	4.6	25.0	561	
	triblock	28.0	76/24	4.0	N/A	6.0	[39]
	triblock	49.8	87/13	11.7	N/A	6.8	
	multiblock	55.3	75/25	22.1	N/A	469	
	multiblock	66.8	87/13	25.2	N/A	59	
Poly(butylene adipate-co-terephthalate) (PBAT)	N/A	N/A	N/A	18.8	30	388	[40]
Poly(butylene succinate) (PBS)	N/A	N/A	N/A	33.3	450	330	[41]
Polypropylene (PP)	N/A	N/A	N/A	34.4	1620	>350	[42]

## References

[1] Bioplastics Market Data. https://www.european-bioplastics.org/market/.

[2] Ho K.-L.G., Pometto A.L., Hinz P.N., Gadea-Rivas A., Briceño J.A., Rojas A. (1999). Field Exposure Study of Polylactic Acid (PLA) Plastic Films in the Banana Fields of Costa Rica. J. Environ. Polym. Degrad..

[3] Rudnik E., Briassoulis D. (2011). Degradation behaviour of poly(lactic acid) films and fibres in soil under Mediterranean field conditions and laboratory simulations testing. Ind. Crops Prod..

[4] Kale G., Auras R., Singh S.P., Narayan R. (2007). Biodegradability of polylactide bottles in real and simulated composting conditions. Polym. Test..

[5] Emadian S.M., Onay T.T., Demirel B. (2017). Biodegradation of bioplastics in natural environments. Waste Manag..

[6] Dilkes-Hoffman L.S., Pratt S., Lant P.A., Laycock B., Al-Salem S.M. (2019). The Role of Biodegradable Plastic in Solving Plastic Solid Waste Accumulation. Plastics to Energy.

[7] Yin S., Tuladhar R., Shi F., Shanks R.A., Combe M., Collister T. (2015). Mechanical reprocessing of polyolefin waste: A review. Polym. Eng. Sci..

[8] Beltrán F.R., Lorenzo V., Acosta J., de la Orden M.U., Martínez Urreaga J. (2018). Effect of simulated mechanical recycling processes on the structure and properties of poly(lactic acid). J. Environ. Manag..

[9] Żenkiewicz M., Richert J., Rytlewski P., Moraczewski K., Stepczyńska M., Karasiewicz T. (2009). Characterisation of multi-extruded poly(lactic acid). Polym. Test..

[10] Pillin I., Montrelay N., Bourmaud A., Grohens Y. (2008). Effect of thermo mechanical cycles on the physico-chemical properties of PLA. Polym. Degrad. Stab..

[11] Zhao P., Rao C., Gu F., Sharmin N., Fu J. (2018). Close-looped recycling of polylactic acid used in 3D printing: An experimental investigation and life cycle assessment. J. Clean. Prod..

[12] Al-Itry R., Lamnawar K., Maazouz A. (2014). Reactive extrusion of PLA, PBAT with a multi-functional epoxide: Physico-chemical and rheological properties. Eur. Polym. J..

[13] Jaszkiewicz A., Bledzki A.K., Duda A., Galeski A., Franciszczak P. (2014). Investigation of Processability of Chain-Extended Polylactides During Melt Processing—Compounding Conditions and Polymer Molecular Structure. Macromol. Mater. Eng..

[14] Sirisinha K., Samana K. (2021). Improvement of melt stability and degradation efficiency of poly (lactic acid) by using phosphite. J. Appl. Polym. Sci..

[15] Luo J., Meng X., Gong W., Jiang Z., Xin Z. (2019). Improving the stability and ductility of polylactic acid via phosphite functional polysilsesquioxane. RSC Adv..

[16] Botta L., Scaffaro R., Sutera F., Mistretta M.C. (2018). Reprocessing of PLA/Graphene Nanoplatelets Nanocomposites. Polymers.

[17] Meng Q., Heuzey M.-C., Carreau P.J. (2012). Control of thermal degradation of polylactide/clay nanocomposites during melt processing by chain extension reaction. Polym. Degrad. Stab..

[18] Araújo A., Botelho G., Oliveira M., Machado A.V. (2014). Influence of clay organic modifier on the thermal-stability of PLA based nanocomposites. Appl. Clay Sci..

[19] Fukushima K., Tabuani D., Arena M., Gennari M., Camino G. (2013). Effect of clay type and loading on thermal, mechanical properties and biodegradation of poly(lactic acid) nanocomposites. React. Funct. Polym..

[20] Oh J.K. (2011). Polylactide (PLA)-based amphiphilic block copolymers: Synthesis, self-assembly, and biomedical applications. Soft Matter.

[21] Stefaniak K., Masek A. (2021). Green Copolymers Based on Poly(Lactic Acid)—Short Review. Materials.

[22] Zhang J., Xu J., Wang H., Jin W., Li J. (2009). Synthesis of multiblock thermoplastic elastomers based on biodegradable poly (lactic acid) and polycaprolactone. Mater. Sci. Eng. C.

[23] Buwalda S.J., Dijkstra P.J., Feijen J. (2012). Poly(ethylene glycol)–poly(L-lactide) star block copolymer hydrogels crosslinked by metal–ligand coordination. J. Polym. Sci. Part A Polym. Chem..

[24] Koosomsuan W., Phinyocheep P., Sirisinha K. (2018). Facile melt processing technique for the preparation of super ductile PLA–PEG multiblock copolymers: The roles of catalyst and antioxidant loadings. Polym. Degrad. Stab..

[25] Luangkachao J., Sirisinha K. (2020). Role of Sn-based and Ti-based catalysts on melt copolymerization of PLA-Polyols. IOP Conf. Ser. Mater. Sci. Eng..

[26] Han L., Yu C., Zhou J., Shan G., Bao Y., Yun X., Dong T., Pan P. (2016). Enantiomeric blends of high-molecular-weight poly(lactic acid)/poly(ethylene glycol) triblock copolymers: Enhanced stereocomplexation and thermomechanical properties. Polymer.

[27] Ding Y., Feng W., Lu B., Wang P., Wang G., Ji J. (2018). PLA-PEG-PLA tri-block copolymers: Effective compatibilizers for promotion of the interfacial structure and mechanical properties of PLA/PBAT blends. Polymer.

[28] Ljungberg N., Wesslén B. (2003). Tributyl citrate oligomers as plasticizers for poly (lactic acid): Thermo-mechanical film properties and aging. Polymer.

[29] Jia Z., Tan J., Han C., Yang Y., Dong L. (2009). Poly(ethylene glycol-co-propylene glycol) as a macromolecular plasticizing agent for polylactide: Thermomechanical properties and aging. J. Appl. Polym. Sci..

[30] Gao F. (2004). Clay/polymer composites: The story. Mater. Today.

[31] Nofar M., Salehiyan R., Ray S.S. (2021). Influence of nanoparticles and their selective localization on the structure and properties of polylactide-based blend nanocomposites. Compos. Part B Eng..

[32] Alexandre M., Dubois P. (2000). Polymer-layered silicate nanocomposites: Preparation, properties and uses of a new class of materials. Mater. Sci. Eng. R Rep..

[33] Gómez M., Palza H., Quijada R. (2016). Influence of Organically-Modified Montmorillonite and Synthesized Layered Silica Nanoparticles on the Properties of Polypropylene and Polyamide-6 Nanocomposites. Polymers.

[34] Krikorian V., Pochan D.J. (2003). Poly (l-Lactic Acid)/Layered Silicate Nanocomposite:  Fabrication, Characterization, and Properties. Chem. Mater..

[35] Franco-Urquiza E.A., Cailloux J., Santana O., Maspoch M.L., Velazquez Infante J.C. (2015). The Influence of the Clay Particles on the Mechanical Properties and Fracture Behavior of PLA/o-MMT Composite Films. Adv. Polym. Technol..

[36] Haji Abdolrsaouli M., Babaei A., Kaschta J., Nazockdat H. (2019). Polylactide/organoclay nanocomposites: The effect of organoclay types on the structure development and the kinetic of cold crystallization. J. Vinyl Addit. Technol..

[37] Maglio G., Migliozzi A., Palumbo R. (2003). Thermal properties of di- and triblock copolymers of poly(l-lactide) with poly(oxyethylene) or poly(ε-caprolactone). Polymer.

[38] Mei T., Zhu Y., Ma T., He T., Li L., Wei C., Xu K. (2014). Synthesis, characterization, and biocompatibility of alternating block polyurethanes based on PLA and PEG. J. Biomed. Mater. Res. Part A.

[39] Wan Y., Chen W., Yang J., Bei J., Wang S. (2003). Biodegradable poly(l-lactide)-poly(ethylene glycol) multiblock copolymer: Synthesis and evaluation of cell affinity. Biomaterials.

[40] Sirisinha K., Somboon W. (2012). Melt characteristics, mechanical, and thermal properties of blown film from modified blends of poly(butylene adipate-co-terephthalate) and poly(lactide). J. Appl. Polym. Sci..

[41] Kajornprai T., Suttiruengwong S., Sirisinha K. (2021). Manipulating Crystallization for Simultaneous Improvement of Impact Strength and Heat Resistance of Plasticized Poly(l-lactic acid) and Poly(butylene succinate) Blends. Polymers.

[42] Premphet K., Horanont P. (2001). Improving performance of polypropylene through combined use of calciuum carbonate and metallocene-produced impact modifier. Polym. -Plast. Technol. Eng..

[43] Von Burkersroda F., Schedl L., Göpferich A. (2002). Why degradable polymers undergo surface erosion or bulk erosion. Biomaterials.

[44] Xu G., Chen S., Yan X., Yang C., Chen Z. (2016). Synthesis and Hydrophilic Performance of Poly(Lactic Acid)-Poly(Ethylene Glycol) Block Copolymers. Am. J. Anal. Chem..

[45] Sheng Y., Yuan Y., Liu C., Tao X., Shan X., Xu F. (2009). In vitro macrophage uptake and in vivo biodistribution of PLA–PEG nanoparticles loaded with hemoglobin as blood substitutes: Effect of PEG content. J. Mater. Sci. Mater. Med..

[46] Paul M.A., Delcourt C., Alexandre M., Degée P., Monteverde F., Dubois P. (2005). Polylactide/montmorillonite nanocomposites: Study of the hydrolytic degradation. Polym. Degrad. Stab..

